# Glass Transition Prediction of Binary Copolymers Across Large Chemical Spaces Using Machine Learning and Physics-Based Modeling

**DOI:** 10.3390/polym18141727

**Published:** 2026-07-14

**Authors:** Manav Bhati, Mohammad Atif Faiz Afzal, Alex K. Chew, Andrea R. Browning, Mathew D. Halls

**Affiliations:** 1 Schrödinger Inc., Portland, OR 97204, USA; 2 Schrödinger Inc., New York, NY 10036, USA; 3 Schrödinger Inc., San Diego, CA 92121, USA

**Keywords:** glass transition temperature (Tg), copolymers, polymer informatics, machine learning, formulation modeling, molecular dynamics (MD), high-throughput screening, composition–property relationships, elastic properties, Young’s modulus, shear modulus, bulk modulus

## Abstract

The glass transition temperature (Tg) is a pivotal design parameter for polymer performance across diverse applications, yet its rapid prediction within expansive chemical spaces remains a challenge. We present a machine learning (ML) framework for the high-throughput prediction of Tg in binary copolymers, trained on experimental datasets encompassing both homopolymers and copolymers. We evaluate various ML architectures, including graph-based algorithms, to effectively capture non-linear composition–property relationships. The optimized model achieves high predictive accuracy with an RMSE of ~14K and an R^2^ of ~0.98. Crucially, the framework accounts for the chemical diversity of monomeric units by integrating structural descriptors with molar composition ratios, enabling the model to capture complex dependencies of thermal stability on chemical structure and composition. We validate the model’s robustness using physics-based molecular dynamics (MD) simulations. To showcase the platform’s scalability, we generated a library of approximately 148,000 binary copolymer compositions and predicted their Tg, facilitating the rapid mapping of vast design spaces. This extensive virtual library enables the identification of optimal monomer pairings that would be experimentally inaccessible through traditional trial-and-error methods. Through these large-scale exploration studies, we demonstrate the ability to design copolymers for targeted applications, including a specific case study on elastomeric systems. This integrated approach, combining experimental data, ML modeling, and physics-based validation, offers a transformative path for the accelerated discovery and multi-property optimization of functional copolymers.

## 1. Introduction

Copolymers play a central role in modern materials design due to their tunable thermal, mechanical, and functional properties, enabling applications across consumer products, electronics, coatings, elastomers, adhesives, biomedical devices, etc. Compared to homopolymers, they offer enhanced design flexibility through control over chemistry and composition of their monomers, allowing targeted optimization of their properties. The glass transition temperature (Tg) is a critically important polymer property, as it dictates its performance, processability, and suitability for applications. For example, high-Tg copolymers are desirable for electronic and structural applications requiring thermal resistance, whereas low-Tg copolymers are preferred for elastomeric and flexible materials.

The Tg of a copolymer is strongly dependent on the chemical structures and composition of its monomers, and the dependence on composition could be linear, convex, concave or any other shape, making the prediction challenging [1,2,3]. Experimental determination of Tg is time and resource intensive, limiting its applicability for rapid screening. Molecular dynamics (MD) simulations are comparatively more efficient and have proven to be reliable and accurate in Tg determination [4,5]. However, they still remain computationally expensive for exploration of a large chemical space and novel copolymer design.

Machine learning (ML) has emerged as a powerful approach for predicting polymer properties directly from molecular descriptors, offering orders-of-magnitude speedup compared to experiments and simulations [6]. While several ML models have been focused on homopolymer property predictions, as in [7,8,9,10,11], relatively fewer studies focus on copolymers, where composition dependence and chemical diversity introduce additional complexity [12,13]. Recent work on machine learning for formulations has highlighted models based on graph-based approaches outperforming other methods for the rapid and accurate prediction of various properties of mixtures of chemicals across a range of applications [14].

In this work, we leverage the graph-based algorithms to develop a robust, transferable and accurate ML model for predicting Tg of copolymers. Our model is trained on a curated dataset of 666 experimental data points encompassing both homopolymers and copolymers. The model captures chemical diversity and composition dependence, enabling reliable Tg prediction across a broad copolymer design space. We validate the model’s performance quantitatively against MD simulations and demonstrate its practical utility by screening a massive library of 148,000 copolymer compositions. Finally, we present a case study on elastomeric systems to illustrate how our approach enables rapid identification of high-performance copolymers and provides a foundation for inverse design of functional materials.

## 2. Methods

### 2.1. Experimental Dataset

**Figure 1:**
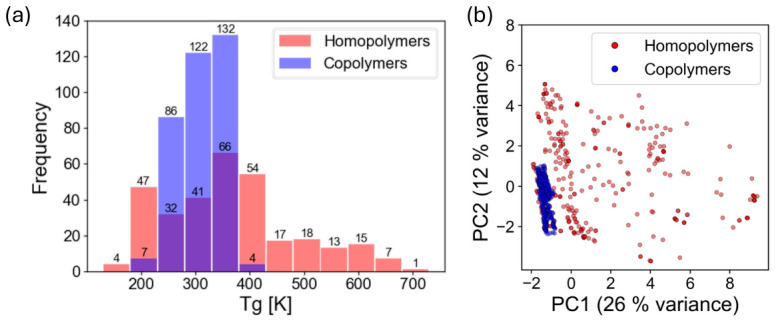
(**a**) Histogram showing the Tg distributions for homopolymers and copolymers. Each bin corresponds to a temperature of 50 K and the number of polymers representing each bin is mentioned on its top. (**b**) Scatter plot showing the distribution on all datapoints from a 2D Principal Component Analysis (PCA). The copolymers (blue) are concentrated and homopolymers (red) are more spread out.

Two datasets of experimental Tg were collected in this work: (1) 301 datapoints from Bicerano et al. [15] and (2) 365 datapoints from Penzel et al. [2]. The Bicerano dataset consists of unique homopolymers. The Penzel dataset has 14 unique homopolymers and 351 random binary copolymers (A-random-B) generated from these homopolymers. The homopolymers A, B are methyl acrylate, ethyl acrylate, *n*-butyl acrylate, isobutyl acrylate, *t*-butyl acrylate, 2-ethyl hexylacrylate, methyl methacrylate, ethyl methacrylate, *n*-butyl methacrylate, isobutyl methacrylate, *t*-butyl methacrylate, acrylonitrile, styrene and vinyl chloride. The monomer composition ranged from 0% to 100% in the steps of 20%, with (100%, 0%) representing the 14 homopolymers. The combined dataset comprises 315 homopolymers and 351 binary copolymers. Figure 1 shows the Tg distribution of homopolymers and copolymers with (a) histogram and (b) scatter plot from Principal Component Analysis (PCA). The histogram shows a 550 K and 250 K spread in Tg values for homopolymers and copolymers, respectively. Since most copolymers in the combined dataset have acrylate-based chemistry, their Tg distribution is much narrower compared to homopolymers which have a wide variety of chemical space. For the PCA, MACCS keys were used as featurizers. From a set of 668 features per data point, two uncorrelated variables, Principal Component 1 and 2 (PC1 and PC2), are derived as linear combinations of the features. PCA is useful for visualizing complex multi-dimensional datasets in a 2D plot. Consistent with histogram, we observe that copolymers are highly concentrated in a specific region of PCA plot, whereas homopolymers are more scattered, representing chemical diversity.

### 2.2. Machine Learning Models

We trained formulation–property relationships with machine learning models as described in Ref. [14]. Input ingredients were represented as Simplified Molecular Input Line Entry System (SMILES), and they were featurized for either descriptor-based or graph-based models. For descriptor-based models, ingredients can be featurized with RDKit descriptors, Matminer descriptors [16], Morgan fingerprints, or MACCS Keys fingerprints. These pre-defined descriptors were then weighted by the ingredients’ composition and aggregated by computing the average, minimum, maximum, standard deviation, and median as described in Equation (1).

(1) $$f=[\mathrm{mean}\left(z_{i}\right),\min\left(z_{i}\right),\max\left(z_{i}\right),\mathrm{std}\left(z_{i}\right),\mathrm{median}\left(z_{i}\right)]$$

where $z_{i}=c_{i}x_{i}$ represent the compositionally weighted features of the monomers i=1,2. The composition $c_{i}$ is the mole fraction of monomer *i*, and the sum of compositions is unity for a binary copolymer system. The final output feature vector f is a concatenation of all aggregation operations. Feature vector f was used as input to machine learning models, such as elastic net, random forest, extreme gradient boosting (XGBoost) [17], support vector machines, and deep neural networks. Elastic net, random forest, and support vector regression were implemented using the scikit-learn package (Version 1.2.1) [18], XGBoost was implemented with the xgboost package (Version 1.7.4) [17], and deep neural networks were implemented in PyTorch (Version 2.0.1) [19]. For graph-based models, ingredients were represented as disconnected graphs, where each ingredients’ atoms and bonds were represented as nodes and edges, respectively. Each node is represented by 75 atomic features and the composition of each ingredient, which were used for graph convolution operations to aggregate features based on neighboring nodes. Ten graph-based models were evaluated: Graph Convolutional Neural Network (GCN) [20], Pytorch version of GCN (TorchGraphConv) [21], TopK [22], GraphSAGE [23], Graph Isomorphism Network (GIN) [24], Self-Attention Graph Pooling (SAGPool) [25], EdgePool [26], GlobalAttention [24], Set2Set [27], and SortPool [28]. Additional details of descriptors-based and graph-based models can be found in our previous work [14].

During model training, the dataset was split into 90% train and 10% test sets with an out-of-sample procedure, where unique formulations were iteratively introduced into the training set until it reached 90% of the dataset, while the remaining 10% of the data was placed in the test set. Out-of-sample splitting provides a more realistic accuracy as compared to random splitting, because random splitting may lead to the same mixture of ingredients appearing in both train and test sets but at different compositions that lead to overzealous prediction accuracies [29]. To implement out-of-sample splitting, we first canonicalized each monomer SMILES string in the dataset to ensure uniqueness, then sorted and aggregated the SMILES of monomers into a single string to create a unique identifier for copolymer systems. These unique identifiers were used to iteratively populate the training set by adding groups of the same copolymer until the training and testing split criteria is satisfied. If the split criteria is not satisfied based on the order of how the groups were added, the algorithm randomly shuffles the groups and repeats the process until the split criteria is satisfied. The best featurizer and models were selected using DeepAutoQSAR’s Bayesian optimization approach [30], which determines the best-performing model based on its performance during five-fold cross validation (5-CV). In 5-CV, the training set is partitioned into five sets using the same out-of-sample splitting procedure, where for each fold, one set is left out as the validation set and the remaining sets are used to train the model; this procedure is repeated five times until all the training data instances are within the left-out set exactly once. After 30 cycles of Bayesian optimization, the mean and standard deviation of the 5-CV coefficient of determination (R^2^) for each candidate architecture are used to compute the lower bound of a 90% confidence interval, which serves as the selection score. The top three architectures with the highest scores are selected to form the final ensemble model, whose predictions are the average across all ensemble members and whose prediction uncertainty is estimated by the standard deviation of the predictions. The details of the parameters of the top three model architectures for the best ensemble model are provided in Table S1 of the Supplementary Information.

### 2.3. Physics-Based Simulations

We utilize physics-based all atom molecular dynamics (MD) simulation to obtain Tg and elastic constants of copolymers. We use Schrödinger’s Materials Science Suite (MSS), version 2024-3 [31] along with OPLS4 force field parameters [32], running on a GPU-based Desmond MD engine [33,34], for all simulations.

#### 2.3.1. MD Simulation Protocol for Tg

The workflow for Tg calculation involves generating polymers, packing them in a simulation cell, equilibrating the cell with molecular dynamics (MD) simulations and then performing MD over a temperature range from high to low to obtain Tg. First, the SMILES strings were used to generate polymers with MSS Polymer Builder. The builder generates 3D polymer structures with random monomer arrangement (for copolymers) at the desired composition. Each polymer chain was of a similar size, with ~2000 atoms. Using MSS’s Disordered System Builder, six chains for each polymer were packed in a simulation cell at an initial density of 0.5 g/cm^3^ to create an amorphous bulk polymer system of ~12,000 atoms. For each polymer, three such replicates were created to obtain better statistical accuracy on Tg calculation. The bulk systems were equilibrated using MSS’s multistage molecular dynamics (MD) workflow. The workflow starts with a quick Brownian minimization for 150 ps, followed by an MD simulation in NVT ensemble (constant moles, volume, and temperature) at 500 K for 500 ps. Then, a compressive MD simulation is performed in NPT ensemble (constant moles, pressure, and temperature) at pressure and temperature of 1000 bar and 400 K for 1 ns, followed by a 17 ns NPT MD simulation at 1 atm and 298 K. Finally, a 21 ns NVT MD is run at 298 K. The MD equilibrated systems were then passed to a Tg simulation workflow, where sequential MD simulations were performed in NPT ensemble at a pressure of 1 atm. The temperature was lowered from 900 K to 100 K in the steps of 10 K and a 5 ns MD was run at each temperature step to determine polymer density as a function of temperature. A hyperbolic fit was applied on the resulting density-temperature data to estimate the Tg. For certain polymers with high Tg, the high-temperature density data were insufficient to generate a reliable hyperbolic fit. To address this, extended temperature ranges were employed for high-Tg systems while maintaining the same 10 K step size. Specifically, polymers with Tg > 450 K were simulated over a temperature range of 1100–200 K, whereas polymers with Tg > 550 K were simulated over a range of 1300–200 K. More details on Tg calculations can be found in Afzal et al. [4].

#### 2.3.2. MD Simulation Protocol for Elastic Constants

The same MD equilibrated systems used as an input to the Tg calculation were used here as an input. The systems were strained uniaxially in three directions (x, y, and z) with six incremental strain steps, each with a magnitude of 0.002. Both tensile and compressive strains were applied uniaxially while maintaining constant lateral dimensions. In addition, the systems were also strained biaxially (pair-wise) with the same number of strain steps and magnitude. MD simulations were performed at 300 K for 500 ps for all strained configurations to calculate energies and stresses, which were subsequently used to determine the elastic constants. The full 6×6 elastic stiffness tensor was obtained in Voigt notation and inverted to yield the compliance matrix, from which the Young’s modulus, shear modulus and bulk modulus were calculated. Three independent replicate simulations were performed for each elastic constant calculation to ensure statistical reliability.

## 3. Results

### 3.1. Machine Learning Models for Tg of Copolymers

**Figure 2:**
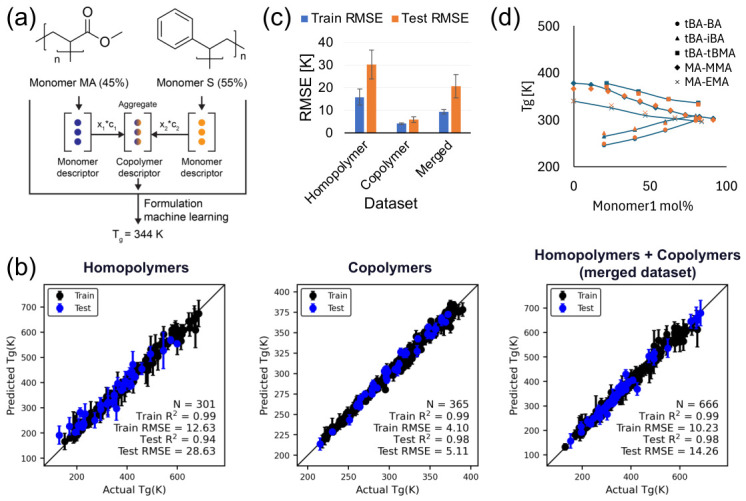
(**a**) Workflow for making predictions on Tg of copolymers. (**b**) Performance of best ML models trained on homopolymers, copolymers, and merged dataset of both. Total number of datapoints, test and train R^2^ and RMSE are shown on the parity plots. (**c**) Average test and train RMSE of the six models trained with random test:train splits on the three datasets. (**d**) Comparison of ML-predicted and experimental composition-dependent Tg curves for tBA-BA (*t*-butyl acrylate—*n*-butyl acrylate), tBA-iBA (*t*-butyl acrylate—isobutyl acrylate), tBA-tBMA (*t*-butyl acrylate—*t*-butyl methacrylate), MA-MMA (methyl acrylate—methyl methacrylate), and MA-EMA (methyl acrylate—ethyl methacrylate) copolymers. The composition on the x-axis represents the mol% of the first monomer in each pair. The blue datapoints with solid lines represent experimental Tg data and orange datapoints without lines represent ML-predicted Tg data, which is from the test set of the best ML model on the merged dataset. The x-axis indicates the mole percentage of the first monomer in each pair.

The workflow for determining Tg for copolymers using ML is described in Figure 2a, where the descriptors for each type of monomers are featurized and then a weighted aggregation is performed based on the monomer composition in the copolymer to obtain copolymer descriptors. ML models are then trained on these descriptors to establish the structure–property relationship and Tg is obtained using the trained models. For single component polymers (homopolymers), the monomer descriptors are directly used.

First, we trained both the datasets separately, and for each, six independent models were trained based on random seeds. As shown in Figure 2b, the training and test results look promising for both datasets. The parity plots show the performance of best models for each dataset. The test RMSE of 28.63 K for homopolymer dataset is higher than that of copolymer dataset (5.11 K) because of its higher chemical diversity. The weighted aggregation described in Figure 2a improves prediction accuracy compared to the models without this step. This is further illustrated in Figure S1 in the Supplementary Information, where two approaches are compared: (a) training ML models using concatenated SMILES, and (b) using the workflow described above. The first approach results in a test RMSE of 19.64 K, whereas the second approach achieves a significantly lower RMSE of 5.11 K.

We also combined the two dataset and trained ML models on the merged dataset. The best-performing model achieved a test RMSE of 14.26 K, with the predictions closely aligning with the parity line and showing no significant outliers. Figure 2c shows the average RMSEs from six independent models trained on each dataset with different test:train split. Overall, models trained on the merged dataset perform better, as they capture both chemical diversity and composition dependence, and will therefore be used in the following sections. Furthermore, using the test data of best-performing ML model presented in Figure 2b, we compared the ML-predicted and experimental composition-dependent Tg curves for selected copolymers in Figure 2d. The close agreement between these curves demonstrates the ability of the ML model to accurately predict Tg values consistent with experimental observations.

### 3.2. Enumeration of Copolymers Dataset and MD Validation of a Subset

**Figure 3:**
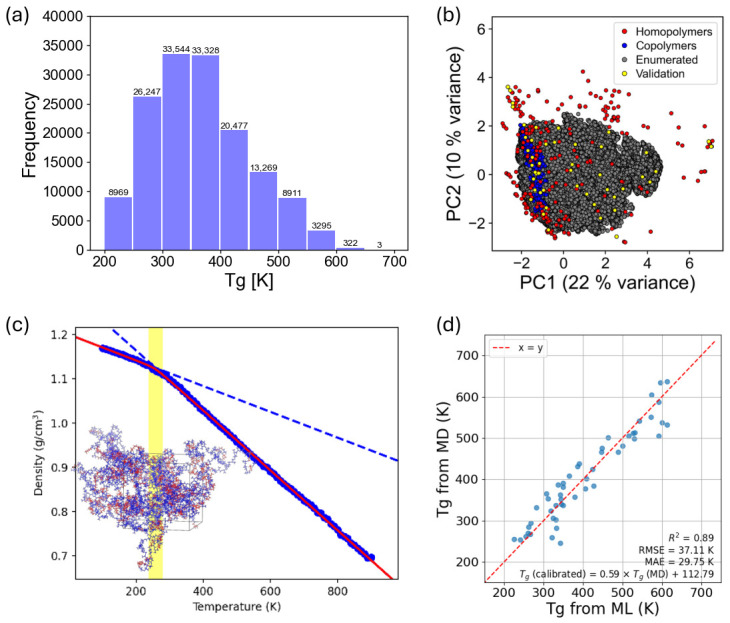
(**a**) The distribution of Tg predicted using the merged-dataset-trained ML model for the enumerated copolymers. (**b**) PCA of all the enumerated copolymers along with the original training data from Figure 1b and copolymers selected for validation of Tg ML models using MD simulations. (**c**) Example of a hyperbolic fit to determine Tg from the MD. The blue data points represent the simulation results used to fit the hyperbolic function, shown as the solid red curve. The two asymptotes of the fitted hyperbola are indicated by blue dashed lines, while the non-asymptotic transition region is highlighted in yellow. The glass transition temperature is defined as the temperature corresponding to the intersection of the two asymptotes. The inset contains the final MD snapshot of the system. The blue and red colors in the inset structure represent the two monomers of the binary copolymer. (**d**) The results from MD are calibrated to compare with the ML predicted Tg’s.

The set of 315 unique monomers was enumerated to generate binary copolymers (A-random-B), resulting in 49,455 copolymers. For each monomer pair, the composition of A and B was varied in weight ratios of 25:75, 50:50 and 75:25. This expanded the dataset, resulting in a total of 148,365 unique copolymers. For all these copolymers, we predicted Tg using the developed machine learning model and Tg distribution is shown in the histogram in Figure 3a. The range of Tg values for the enumerated dataset (500 K) is smaller than that of the homopolymer dataset (550 K). Most copolymers have a Tg between 300 and 400 K, making them suitable candidates for applications where polymers need to remain in the glassy state at room temperature, such as electronics, medical devices, etc. The distribution of all copolymers based on PCA is shown in Figure 3b, along with the experimental datapoints for the homopolymer and copolymer datasets (plotted in Figure 1b). The enumerated copolymers fall within the space of homopolymers, as they are created from combinations of two homopolymers, which also explains the smaller range of Tg compared to homopolymers.

Although useful and efficient in predicting Tg, ML models also need to be reliable. To test this, we utilized physics-based MD simulations, which have been shown to predict Tg with good reliability [4]. From the range of Tg values in the enumerated dataset, 10 equally spaced points were selected, and the copolymers closest to these points were identified. A new validation set was then created by varying the monomer composition of these selected copolymers across five weight ratios: 0:100, 25:75, 50:50, 75:25, 100:0. This resulted in a validation set of 50 polymers, including 20 homopolymers (0:100 and 100:0 compositions) and 30 binary copolymers. The 50 polymers are highlighted in Figure 3b. MD simulations, as described in the Methods section, were performed to calculate Tg for the polymers in the validation set. An example of the Tg calculation is shown in Figure 3c, where a hyperbolic fit to the density versus temperature curve yields the Tg. The inset shows a representative copolymer structure from the MD simulation. A comparison between Tg values predicted by the ML model and those obtained from MD simulations is shown in Figure 3d. It is known that MD tends to overestimate Tg for homopolymers [4], and we observe the same trend for copolymers. To address this, a calibration was applied to the MD-derived Tg values to enable direct comparison with the ML predictions. The calibration equation is shown in the plot, where a slope less than one indicates a scale down of Tg values from MD. Overall, the agreement is strong, (R^2^ = 0.89), demonstrating the reliability of the ML models. In addition, the composition-dependent Tg trends for these copolymers, shown in Figure S2 of the Supplementary Information, exhibit close agreement between MD and ML predictions, further highlighting the robustness of the model. This result is particularly significant because the validation polymers were systematically selected from distinct regions of the PCA space, as shown in Figure 3b.

### 3.3. Elastomer Case Study

**Figure 4:**
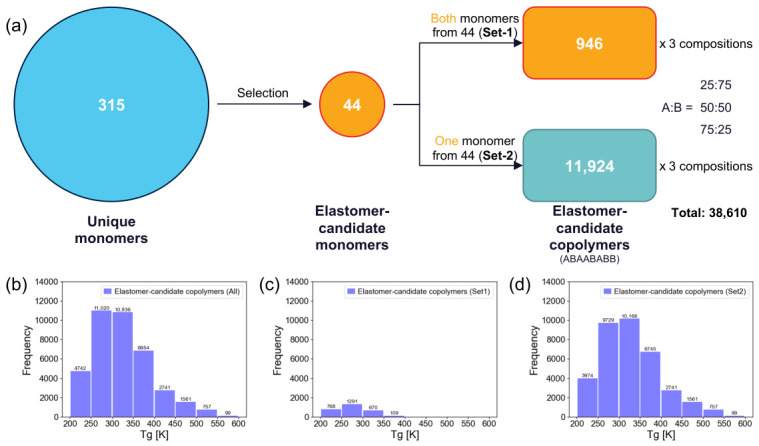
(**a**) Scheme to select elastomer-candidate monomers and create elastomer-candidate copolymers. The distribution of predicted Tg for elastomer-candidate copolymers from (**b**) both Set-1 and Set-2 combined, (**c**) Set-1, and (**d**) Set-2.

We further extended the validation with a case study on elastomers, which are polymers that typically exist in a rubbery state at room temperature and are commonly used in applications such as tires, seals, gloves, etc. Generally, polymers with higher Tg are more rigid compared to those with lower Tg, as they remain in the glassy state at room temperature. Based on this, we validated our ML model by testing whether the high-Tg polymers suggested by the model were mechanically distinct from the low-Tg polymers. To do this, we created a set of possible elastomers (binary copolymers) from the pool of 315 unique monomers, as outlined in Figure 4a. First, 44 monomers were identified based on literature as likely elastomer candidates, and we refer to them as elastomer-candidate monomers [35]. This list is not exhaustive but includes monomers such as butadiene, isoprene, and acrylates which could form elastomers when combined with other monomers. Two enumerations were performed: one in which binary copolymers were created using two elastomer-candidate monomers (Set-1), and another where one monomer came from the elastomer-candidate pool and the other from the remaining pool of unique monomers (Set-2). For each monomer pair, three compositions were created with weight ratios of 25:75, 50:50, and 75:25. This resulted in a total of 38,610 copolymers, which we refer to as elastomer-candidate copolymers, as they contain at least one elastomer-candidate monomer.

The distribution of Tg for these elastomer-candidate copolymers is shown in Figure 4b. Most of these have Tg in the range of 250–350 K and the Set-1 copolymers having no copolymers with Tg higher than 400 K (Figure 4c) as they have both elastomer-candidate monomers. We performed a Z-score analysis [36] to identify the prominent monomers that are present in the lowest Tg copolymers (lowest 10%, 3861 copolymers), as these are most likely to be good elastomers. The Z-score indicates the likelihood of each monomer’s presence in copolymers with the lowest glass transition temperatures (Tg). Consequently, a high Z-score signifies a greater probability that a monomer will appear in low-Tg copolymers. The analysis is included in Figure S3 in the Supplementary Information. Among the monomers with high Z-scores, there are several from Set-1, again suggesting the higher probability of elastomers from Set-1.

**Figure 5:**
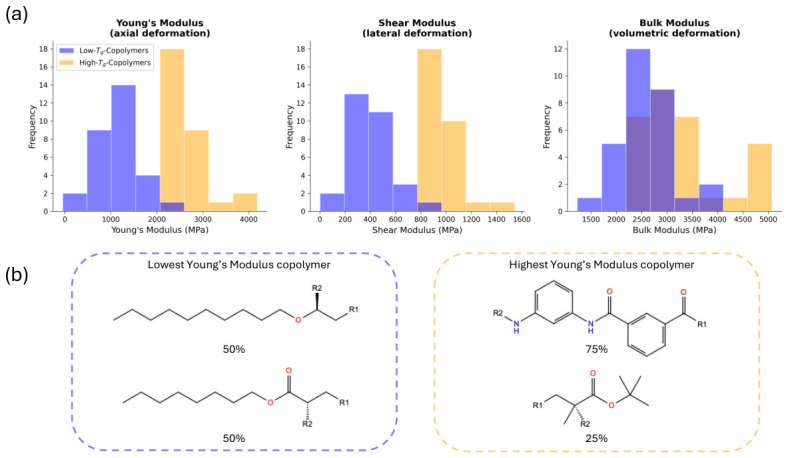
(**a**) The distribution of elastic moduli of low-Tg and high-Tg copolymers. (**b**) Examples of copolymers with lowest and highest Young’s modulus are shown as with monomer ratios.

For the validation, we selected 30 copolymers each from low-Tg and high-Tg regions. The low-Tg copolymers are equally spaced relative to Tg in the range of 200–250 K while the high-Tg copolymers are equally spaced relative in the Tg range of 400–600 K. These temperature ranges have equivalent number of copolymers (~5000 copolymers from Figure 4b). The elastic constant calculations, using the procedure described in Methods section were performed for each of the 60 copolymers and three elastic constants were calculated: Young’s modulus, shear modulus, and bulk modulus. For each of the three moduli, there is a clear distinction in the distribution of values between the low-Tg and high-Tg copolymers as shown in Figure 5. This clearly shows that the low and high-Tg copolymers suggested by ML model are mechanically different, with the high-Tg ones having higher moduli compared to the low-Tg ones. This matches with the general observation and also validates our ML model for Tg.

In future, such ML models will be developed for other polymer properties, like elastic constants, wettability, moisture uptake, etc., and a multiparameter optimization approach could be utilized to obtain optimum polymers that could maximize likelihood of possessing the desired properties.

## 4. Conclusions

The ML model for predicting the Tg of binary copolymers, developed using two experimental Tg datasets, is quantitatively validated against MD simulations and qualitatively assessed through a case study on elastomers. The robustness and predictive accuracy of the model make it well suited for high-throughput Tg screening of large copolymer design spaces, significantly reducing reliance on computationally expensive atomistic simulations and experimental trial-and-error. Future efforts will focus on extending the framework to multicomponent copolymers (ternary and higher-order systems) and integrating Tg prediction within a multi-objective optimization framework targeting simultaneous optimization of thermal, mechanical, and transport properties of polymeric materials.

## Data Availability

The data supporting reported results can be found at https://zenodo.org/records/19242815 (accessed on 5 July 2026).

## Supplementary Materials

The following supporting information can be downloaded at: https://www.mdpi.com/article/10.3390/polym18141727/s1, supplementary information document; experimental_dataset.csv: Experimental Tg dataset for homo- and copolymers used for training ML model; experimental_tg_prediction.csv: ML-predicted Tg for the experimental dataset with corresponding train–test labels; enumerated_copolymer_tg_prediction.csv: Full list of enumerated copolymers with Tg predicted by the trained ML model; enumerated_elastomer_copolymer_tg_prediction.csv: Enumerated elastomer-candidate copolymers with Tg predicted by the trained ML model; md_tg_data.csv: MD- and ML-predicted Tg values for 50 selected copolymers used for validation; md_elastic_constant_data. xlsx: Three elastic constants (averages with standard errors) for each of the 30 high-Tg and low-Tg copolymers calculated from MD simulations. The following tables and figures are provided in the Supporting Information. Table S1: Top-3 models from formulation ML training and the test performance for the best ensemble model on the merged dataset. Figure S1: Comparison of ML models (a) without and (b) with graph-based approach. Figure S2: Comparison of ML-predicted and MD-calibrated composition-dependent Tg curves for copolymers. The copolymers have two monomers: A and B, and the x-axis of each plot has the mol% of monomer A. The A-B pairs are (a) oxytetramethylene-propylene sulfide, (b) *n*-octyl methacrylate-etherimide 9, (c) vinyl chloroacetate-2-methyl-4-chloro styrene, (d) methyl-*p*-xylylene-*n*-butyl α-chloroacrylate, (e) 4-methoxy-2-methyl styrene-quinoline 5, (f) acrylic acid-isobutyl acrylate, (g) oxy-1,4-phenylene-oxy-1,4-phenylene-carbonyl-1,4-phenylene-*n*-butyl methacrylate, (h) etherimide 5-quinoxaline-2,7-diylquinoxaline-7,2-diyl-*p*-terphenyl-4,4’-ylene, (i) quinoline 6-quinoxaline-2,7-diylquinoxaline-7,2-diyl-1,4-phenylene, (j) quinoline 3-*n*-butyl acrylate. Figure S3: Z-score analysis (on left) on the enumerated copolymers with low-Tg (lowest 10%) and selected Set-1 monomers (on right) with high Z-scores.
