# Supplementary material for: Glass Transition Prediction of Binary Copolymers Across Large Chemical Spaces Using Machine Learning and Physics-Based Modeling
Source: Polymers (Basel). 2026 Jul 14;18(14):1727. doi: 10.3390/polym18141727 (PMC13417439; doi:10.3390/polym18141727)
Supplement: Supplementary file 1 [file polymers-18-01727-s001.zip › si.pdf]

Supporting Information for: Glass Transition Prediction of Binary Copolymers Across Large Chemical Spaces Using Machine Learning and Physics-Based Modeling

Manav Bhati,*,† Mohammad Atif Faiz Afzal,*,† Alex K. Chew,‡ Andrea R. Browning,† and Mathew D. Halls¶

†*Schrödinger Inc., Portland, OR, USA*

‡*Schrödinger Inc., New York, NY, USA*

¶*Schrödinger Inc., San Diego, CA, USA*

E-mail: manav.bhati@schrodinger.com; atif.afzal@schrodinger.com

Table S1: Top-3 models from formulation ML training and the test performance for the best ensemble model on the merged dataset.

Parameter	Model 1	Model 2	Model 3	Ensemble
Architecture	GlobalAttention	GlobalAttention	EdgePool	–
Featurizer	Geometric[7]	Geometric	Geometric	–
Layers[1]	[205, 115, 44]	[183, 110]	[154, 46]	–
Dense layer[2]	202	397	155	–
Dropout[3]	0.334	0.212	0.096	–
Epochs[4]	124	88	139	–
Learning rate schedule[5]	linear decay	custom	linear decay	–
Mode	Regression	Regression	Regression	–
Num. features	71	71	71	–
Transformers	Continuous,[8] norm,[9] RCorr/90[10]	log,[11] Continuous, norm, RCorr/90	Continuous, norm, RCorr/90	–
CV R^2 (mean $\pm$ std)[6]	0.936 ± 0.008	0.925 ± 0.020	0.916 ± 0.030	–
Test R^2	–	–	–	0.982
Test RMSE	–	–	–	14.26
Test MAE	–	–	–	9.07
Test Median AE	–	–	–	5.08
Kendall τ	–	–	–	0.911

1. Layers: Comma separated list of neurons used for each layer. For example, Model 1 performs 3 graph convolutions with 205, 115, and 44 number of neurons after each convolution layer.
2. Dense layer: Dense layer indicates the total number of neurons for a fully connected layer.
3. Dropout: Dropout indicates the probability of dropping out nodes for a neural network during model training to prevent overfitting. Dropout is typically between 0.0 to 0.5.
4. Epochs: Epochs mean the number of complete passes of the training dataset used to train the models.
5. Learning rate schedule: Learning rate for neural networks.
6. CV R^2: Cross validation coefficient of determination.
7. Geometric: Geometric means representing formulations as graphs.
8. Continuous: Continuous transformer standardizes all continuous input values so that they have a mean of 0 and standard deviation of 1.
9. norm: Norm transformer standardizes the target value so that they have a mean of 0 and standard deviation of 1.
10. RCorr/90: Removes correlated features that have a Pearson's r correlation of 0.90 or greater.
11. log: Log transformer performs a log transformation on the target value.

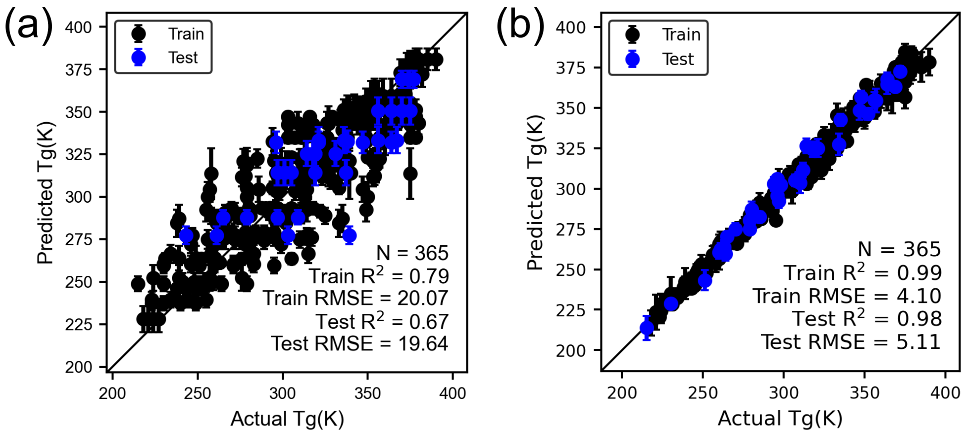

Figure S1: Comparison of ML models (a) without and (b) with graph-based approach

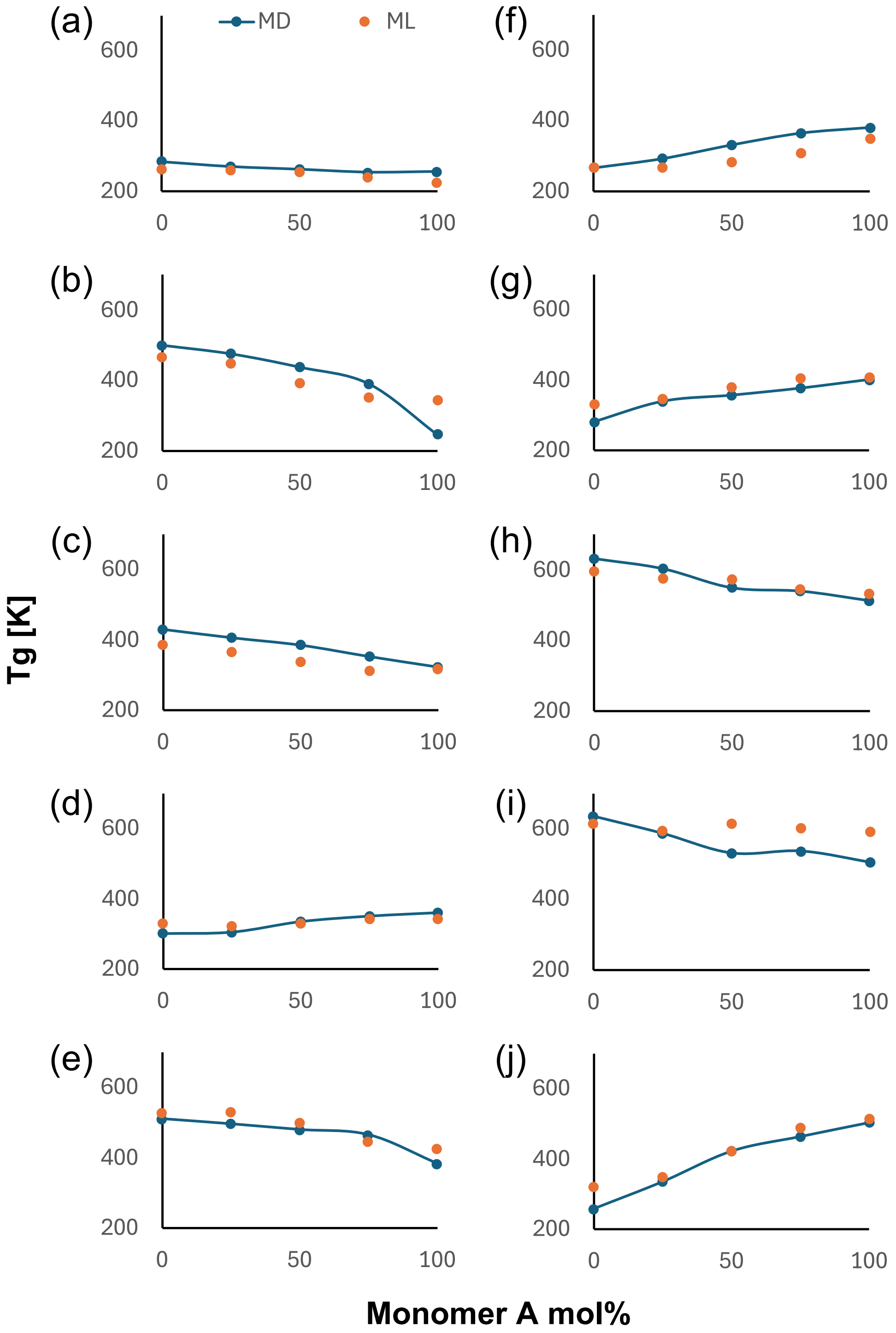

Figure S2: Comparison of ML-predicted and MD-calibrated composition-dependent Tg curves for copolymers. The copolymers have two monomers: A and B, and the x-axis of each plot has the mol% of monomer A. The A - B pairs are (a) oxyte-

tramethylene - propylene sulfide, (b) n-octyl methacrylate - etherimide 9, (c) vinyl chloroacetate - 2-methyl-4-chloro styrene, (d) methyl-p-xylylene - n-butyl a-chloroacrylate, (e) 4-methoxy-2-methyl styrene - quinoline 5, (f) acrylic acid - isobutyl acrylate, (g) oxy-1,4-phenylene-oxy-1,4-phenylene-carbonyl-1,4-phenylene - n-butyl methacrylate, (h) etherimide 5 - quinoxaline-2,7-diylquinoxaline-7,2-diyl-p-terphenyl-4,4'-ylene, (i) quinoline 6 - quinoxaline-2,7-diylquinoxaline-7,2-diyl-1,4-phenylene, (j) quinoline 3 - n-butyl acrylate.

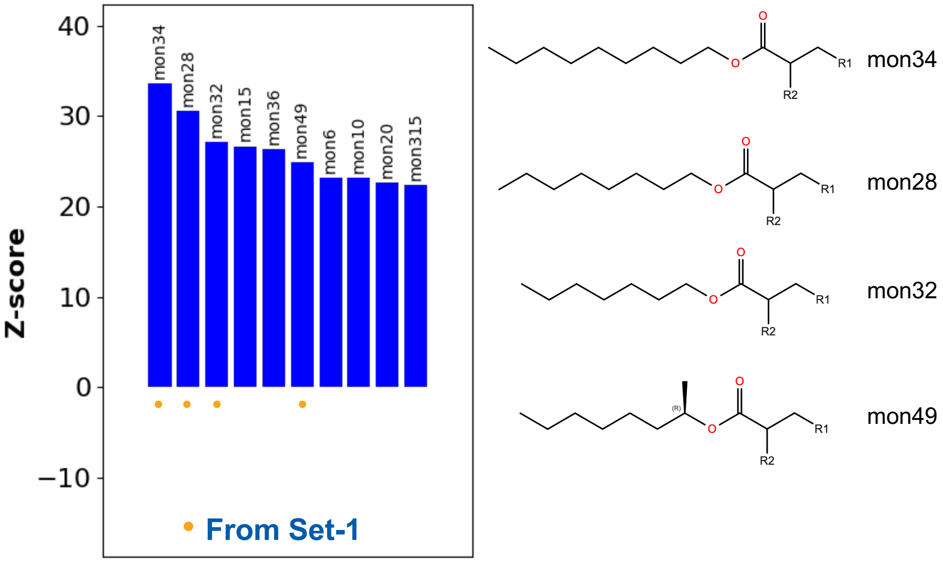

Figure S3: Z-score analysis (on left) on the enumerated copolymers with low-Tg (lowest 10%) and selected Set-1 monomers (on right) with high Z-scores
